# Supplementary material for: Reconciling Mining with the Conservation of Cave Biodiversity: A Quantitative Baseline to Help Establish Conservation Priorities
Source: PLoS One. 2016 Dec 20;11(12):e0168348. doi: 10.1371/journal.pone.0168348 (PMC5173368; doi:10.1371/journal.pone.0168348)
Supplement: S1 Dataset — (ZIP) [file pone.0168348.s002.zip › Taxa/Serra Norte/SN_2013/N4E/Lista N4E-26.pdf]

CAVIDADE N4E-0026

Classe	Ordem	Fam/Outros	Gên/Outros	Espécie	S-U	U-S	Zon.
Annelida	Oligochaeta			sp.	6		P/A
Arachnida	Acari	(Oribatida)		jovem		X	
Arachnida	Acari	Argasidae	*Ornithodoros*	sp.1	X		
Arachnida	Acari	Eupodidae		sp.		X	
Arachnida	Acari	Ixodidae		jovem	X		P
Arachnida	Acari	Mesostigmata		jovem	X		P
Arachnida	Acari	Prostigmata		sp.49	X		P/A
Arachnida	Acari	Prostigmata		sp.53	X		E/P/A
Arachnida	Acari	Trombiculidae		sp.39	X		P
Arachnida	Acari	Trombiculidae		sp.41	X		A
Arachnida	Amblypygi	Phrynidae	*Heterophrynus*	*longicornis*	2	X	A
Arachnida	Araneae	Araneidae		jovem	X	X	P
Arachnida	Araneae	Caponiidae		jovem	X		P
Arachnida	Araneae	Corinnidae	*Creugas*	sp.1	2		
Arachnida	Araneae	Ctenidae		jovem		1	
Arachnida	Araneae	Filistatidae		sp.1	X		E/P
Arachnida	Araneae	Ochyroceratidae	*Ochyrocera*	sp.1	X	X	E/P/A
Arachnida	Araneae	Ochyroceratidae	*Speocera*	sp.1	X	X	E/P/A
Arachnida	Araneae	Oonopidae	aff. *Xyccarph*	sp.2	X		P/A
Arachnida	Araneae	Pholcidae	*Leptopholcus*	sp.1	X		E/P/A
Arachnida	Araneae	Pholcidae	*Mesabolivar*	sp.1	X	X	E/P/A
Arachnida	Araneae	Pholcidae	Ninetinae	sp.1	X		E/P/A
Arachnida	Araneae	Salticidae		jovem	X	X	E/P
Arachnida	Araneae	Scytodidae	*Scytodes*	*eleonorae*	6		P
Arachnida	Araneae	Symphytognathidae	*Anapistula*	sp.1	X		P/A
Arachnida	Araneae	Theridiidae	*Theridion*	sp.		X	
Arachnida	Araneae	Theridiosomatidae	*Plato*	sp.1	X	X	E/P/A
Arachnida	Araneae	Uloboridae		jovem	X		P
Arachnida	Opiliones	Cosmetidae	*Roquettea*	*singularis*	1		
Arachnida	Opiliones	Escadabiidae		sp.2	X	X	A
Arachnida	Opiliones	Manaosbiidae	*Saramacia*	sp.1	5		P
Arachnida	Opiliones	Stygnidae	*Protimesius*	*gracilis*	19	X	E/P
Arachnida	Opiliones	Stygnidae	*Protimesius*	*laevis*	2		
Arachnida	Palpigradi	Eukoeneniidae	*Eukoenenia*	sp.1	X		P
Arachnida	Pseudoscorpiones	Chernetidae	*Spelaeochernes*	sp.1	X	X	E/P/A
Arachnida	Pseudoscorpiones	Chthoniidae	*Pseudochthonius*	sp.1	X	X	E/P/A
Chilopoda	Geophilomorpha	Ballophilidae	*Ityphilus*	sp.2	1		P
Chilopoda	Scolopendromorpha	Scolopocryptopidae	*Dinocryptops*	*miersii*		X	
Chilopoda	Scolopendromorpha	Scolopocryptopidae	*Newportia*	sp.		X	
Chilopoda	Scutigeromorpha	Pselliodidae	*Sphendononema*	*guildingii*	1	X	
Diplopoda	Polydesmida	Chelodesmidae		sp.		X	
Diplopoda	Polydesmida	Pyrgodesmidae		sp.1	X	X	P/A
Entognatha	Collembola	Entomobryidae		sp.1	X		E/P
Entognatha	Collembola	Isotomidae		sp.1	X		P
Entognatha	Collembola	Paronellidae		sp.1	X		P/A
Entognatha	Collembola	Paronellidae		sp.4	X		P
Entognatha	Collembola	Sminthuroidea		sp.2	X	X	A
Entognatha	Diplura	Campodeidae		sp.1	X	X	E
Entognatha	Diplura	Japygidae		sp.1		X	
Entognatha	Diplura	Projapygidae		sp.1		X	
Insecta	Blattodea	Blattellidae		sp.2	1		P
Insecta	Blattodea	Blattellidae		sp.4	1		
Insecta	Blattodea	Blattidae		jovem		X	
Insecta	Blattodea	Polyphagidae		sp.1	3		P
Insecta	Coleoptera	Elateridae		sp.		X	

Insecta	Coleoptera	Scarabaeidae		jovem		X	
Insecta	Coleoptera	Staphylinidae	Pselaphinae	sp.		X	
Insecta	Coleoptera	Tenebrionidae	Alleculinae	jovem		X	
Insecta	Coleoptera			jovem	X	X	E/P/A
Insecta	Diptera	Chironomidae		sp.	X	X	P/A
Insecta	Diptera	Curtonotidae		sp.		X	
Insecta	Diptera	Dolichopodidae		sp.	X		E/P
Insecta	Diptera	Phoridae		sp.		X	
Insecta	Diptera	Psychodidae	*Pintomyia*	*gruta*	X	X	
Insecta	Diptera	Sciaridae		sp.	X		P
Insecta	Diptera	Tipulidae		sp.		X	
Insecta	Heteroptera	Cydnidae		sp.	X	X	P
Insecta	Heteroptera	Reduviidae	Emesinae	sp.	1		P
Insecta	Heteroptera	Reduviidae	*Ploiaria*	sp.		X	
Insecta	Heteroptera	Reduviidae	*Zelurus*	sp.	1	X	
Insecta	Homoptera	Cicadellidae		sp.2	X		P
Insecta	Homoptera	Cixiidae		jovem	X	X	A
Insecta	Hymenoptera	Bethylidae		sp.1	X	X	A
Insecta	Hymenoptera	Braconidae		sp.		X	
Insecta	Hymenoptera	Diapriidae		sp.1	X		A
Insecta	Hymenoptera	Formicidae	*Acromyrmex*	sp.1	X		E/P
Insecta	Hymenoptera	Formicidae	*Anochetus*	sp.1		X	
Insecta	Hymenoptera	Formicidae	*Brachymyrmex*	sp.		X	
Insecta	Hymenoptera	Formicidae	*Camponotus*	*atriceps*		X	
Insecta	Hymenoptera	Formicidae	*Gnamptogenys*	*striatula*		X	
Insecta	Hymenoptera	Formicidae	*Pachycondyla*	*constricta*		X	
Insecta	Hymenoptera	Formicidae	*Pachycondyla*	*harpax*		X	
Insecta	Hymenoptera	Formicidae	*Pachycondyla*	sp.2	X		E/P
Insecta	Hymenoptera	Formicidae	*Paratrechina*	sp.1	X		P
Insecta	Hymenoptera	Formicidae	*Solenopsis*	sp.		X	
Insecta	Hymenoptera	Pompilidae		sp.		X	
Insecta	Isoptera	Rhinotermitidae	*Heterotermes*	sp.	X		P
Insecta	Isoptera	Termitidae	*Nasutitermes*	sp.	X	X	E/P
Insecta	Lepidoptera	Noctuoidea		sp.2	1		E
Insecta	Lepidoptera	Noctuoidea		sp.5	1		A
Insecta	Lepidoptera	Tineoidea		sp.1	X	X	E/P/A
Insecta	Lepidoptera	Tineoidea		sp.2	X		P
Insecta	Lepidoptera	Tineoidea		sp.3	X		P
Insecta	Orthoptera	Gryllidae	Eneopterinae	sp.	1		P
Insecta	Orthoptera	Phalangopsidae	*Paraclodes*	jovem	31		E
Insecta	Orthoptera	Phalangopsidae	*Phalangopsis*	sp.	321	X	P/A
Insecta	Psocoptera	Epipsocidae	*Epipsocus*	sp.2	X		P/A
Insecta	Psocoptera	Psyllipsocidae	*Psyllipsocus*	sp.1	X		P
Insecta	Psocoptera			jovem	X	X	E/P/A
Insecta	Thysanura	Ateluridae	Atelurinae	sp.		X	
Insecta	Thysanura	Nicoletiidae	Nicoletiinae	sp.1		X	
Gastropoda	Pulmonata	Subulinidae	*Allopeas*	sp.		X	
Gastropoda	Pulmonata	Subulinidae	*Lamellaxis*	sp.		X	
Gastropoda	Pulmonata	Subulinidae		sp.4	X		P/A
Gastropoda	Pulmonata	Systrophiidae	*Happia*	sp.	X	X	A
Malacostraca	Isopoda	Armadillidae		sp.1	X	X	E
Malacostraca	Isopoda	Scleropactidae	*Circoniscus*	*buckupi*	X	X	P/A
Amphibia	Anura			sp.	35	1	E/P
Mammalia	Chiroptera	Emballonuridae	*Peropteryx*	*kappleri*	3	3	E/P
Mammalia	Chiroptera	Furipteridae	*Furipterus*	*horrens*	1		P
Mammalia	Chiroptera	Phyllostomidae	*Anoura*	*caudifer*	40		P/A
Mammalia	Chiroptera	Phyllostomidae	*Anoura*	*geoffroyi*	100		P/A
Mammalia	Chiroptera	Phyllostomidae	*Carollia*	*perspicillata*	20		E/P/A

Mammalia	Chiroptera	Phyllostomidae	*Glossophaga*	*soricina*	1		P
Mammalia	Chiroptera	Phyllostomidae	*Lonchophylla*	*thomasi*	1		P
Mammalia	Chiroptera	Phyllostomidae		sp.		40	E/P/A
